# Subacute pacemaker lead migration with cardiac perforation and extracardiac extension into lung parenchyma with associated pneumothorax

**DOI:** 10.1016/j.radcr.2026.01.040

**Published:** 2026-02-19

**Authors:** Alyssa Liang, Vanessa L. Starr, Snehal Adodra, Praveen Anchala, Mohammad H. Madani

**Affiliations:** Santa Clara Valley Medical Center, 751 South Bascom Ave, San Jose, CA 95128, USA

**Keywords:** Pacemaker placement, Pacemaker lead migration, Cardiac perforation, Pneumothorax

## Abstract

Cardiac perforation with extracardiac extension by a pacemaker lead is a rare but serious complication of pacemaker placement. We present a unique case with imaging of a patient who underwent pacemaker placement complicated by subacute migration of the pacemaker lead, resulting in perforation involving interventricular septum, right ventricular apex, pericardium, epicardial fat, pleura and left upper lobe parenchyma with left-sided pneumothorax.

## Introduction

Cardiac perforation and pneumothorax are both rare but potentially life-threatening complications of pacemaker placement, with an approximately 0.4% incidence of cardiac perforation [1] and a 1.3% incidence of pneumothorax [2]. While pneumothorax is more commonly typically seen as a complication of pacemaker placement due to complication of venous access during the pacemaker placement [2,3], cases of right ventricular [4] and right atrial perforation [[5], [6], [7], [8]] due to pacemaker lead migration have been reported with resulting contralateral pneumothorax. Right ventricular perforation may occur as an acute complication within 24 hours of pacemaker placement [4] or as a subacute to chronic complication occurring days to months after pacemaker placement [[9], [10], [11], [12], [13], [14]].

We present a case of a 78-year-old female patient who underwent pacemaker placement complicated by pacemaker lead migration, resulting in the second reported case of subacute right ventricular perforation with extracardiac extension of perforation and left-sided pneumothorax.

## Case presentation

A 78-year-old woman with a history of hypertension, hyperlipidemia, type 2 diabetes, nonischemic cardiomyopathy, sick sinus syndrome, and tachy-brady syndrome, underwent dual-chamber pacemaker placement. She tolerated the procedure well without immediate complications and was discharged 2 days later.

**Fig. 1:**
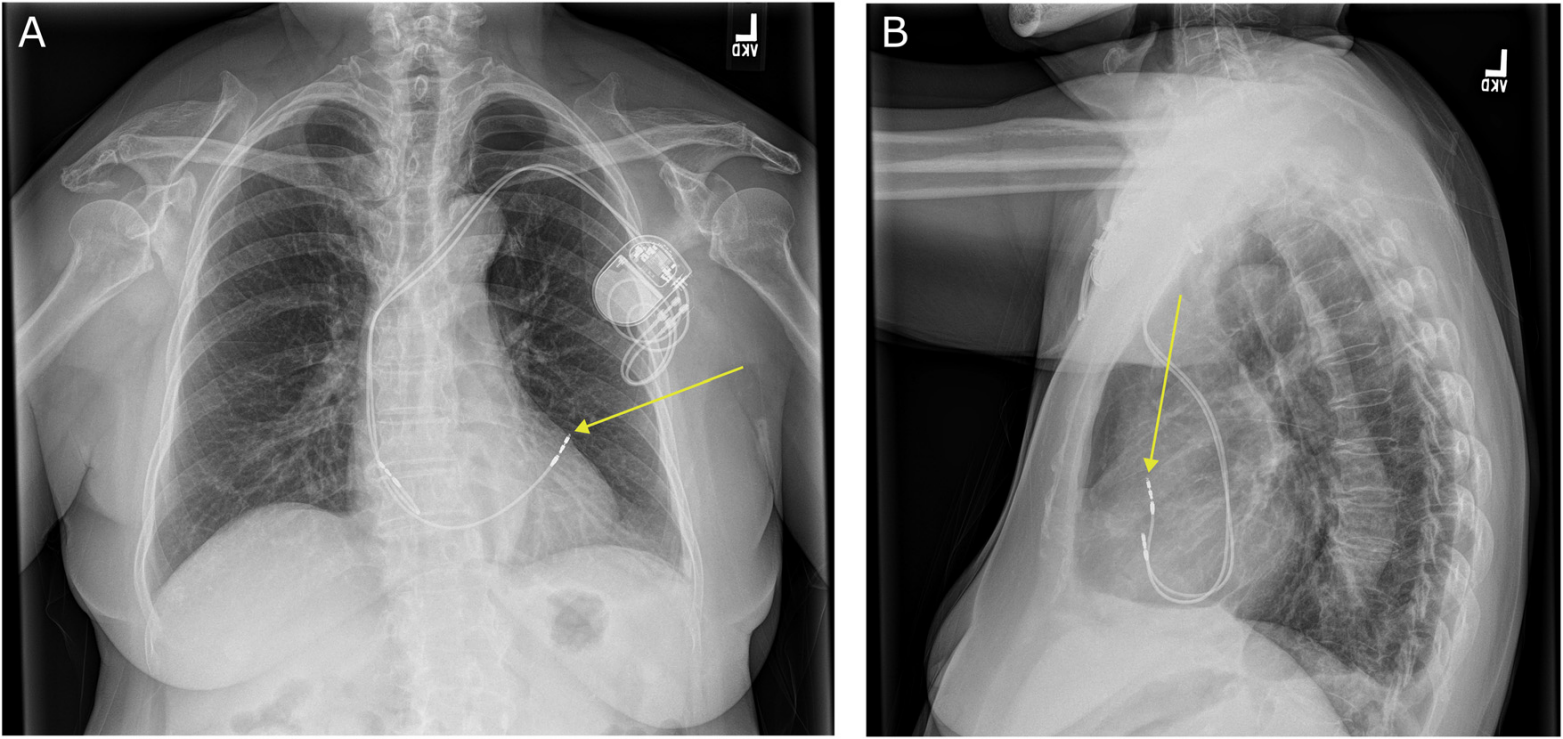
Posteroanterior (A) and lateral (B) radiograph of the chest. The dual chamber pacemaker generator is seen in the left upper chest wall. Pacemaker leads are intact. The atrial lead appears to be in placed in the right atrium. On frontal posteroanterior projection the tip of the right ventricular lead appears to project slightly beyond the cardiac border which is not evident on lateral view (yellow arrow). Small left apical pneumothorax is present.

14 days after pacemaker placement, she presented for follow-up at the cardiac device clinic, and reported a cough and sensation of chest pressure. Device interrogation of the pacemaker revealed a malfunction of the right ventricular lead, with greater than 2000 ohms of impedance and noncapture at the highest threshold in both unipolar and bipolar modes of the pacemaker. There was an initial concern for lead fracture. A chest radiograph showed intact pacemaker leads, but it also showed right ventricular lead migration and a small left pneumothorax (Fig. 1), and she was directed to proceed to the emergency department for further evaluation and management.

In the emergency department, she reported continued cough, pain at the pacemaker placement site, and a sensation of substernal chest pressure, but denied shortness of breath. Her vital signs were within normal limits. Her EKG showed an atrial paced rhythm without other abnormalities. Cardiology was consulted, and recommended admission to the progressive care unit for cardiac monitoring.

**Fig. 2:**
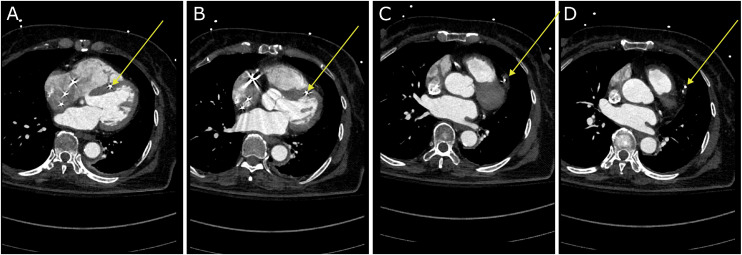
Gated CT angiogram of the chest, showing the right ventricular lead migration through the (A) and (B) interventricular septum towards the apex, (C) through the apex, and (D) into the left upper lobe (yellow arrow).

**Fig. 3:**
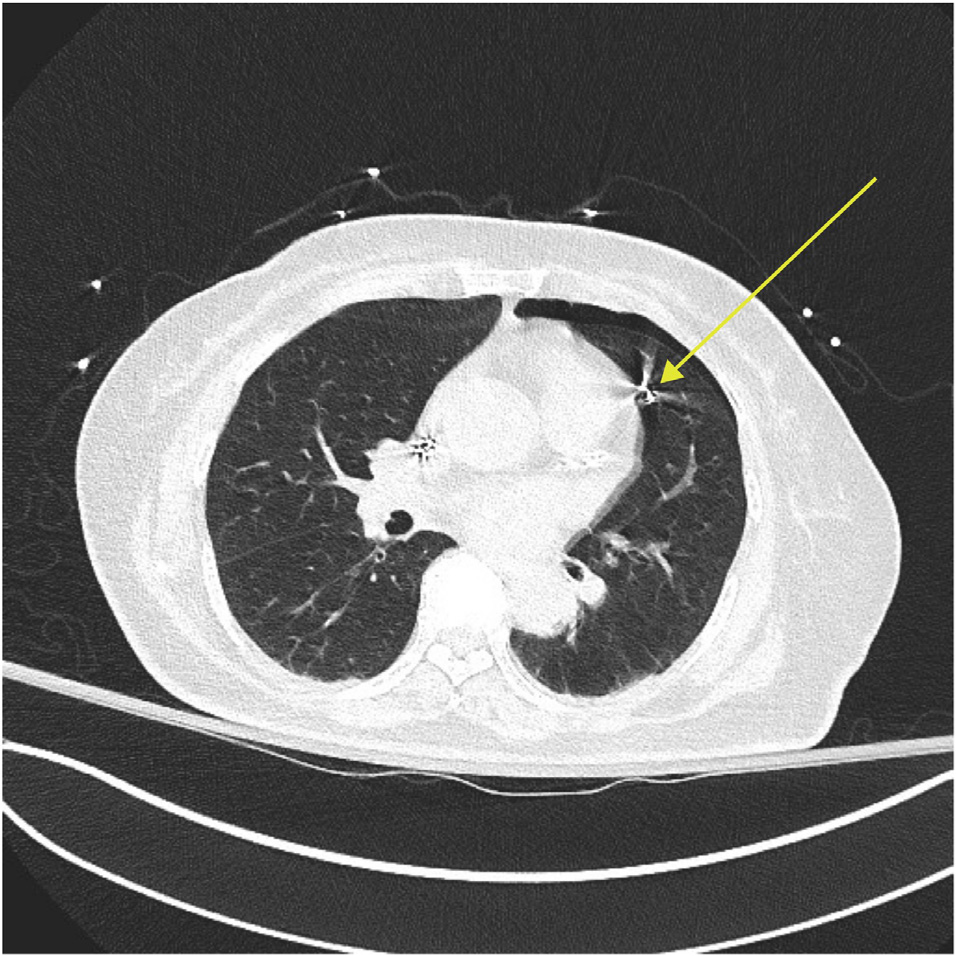
Lung window of the same gated CT angiogram of the chest, showing the left-sided apical pneumothorax. The right ventricular lead can be seen traversing through the pericardium and passing into the left upper lobe of the lung (yellow arrow).

**Fig. 4:**
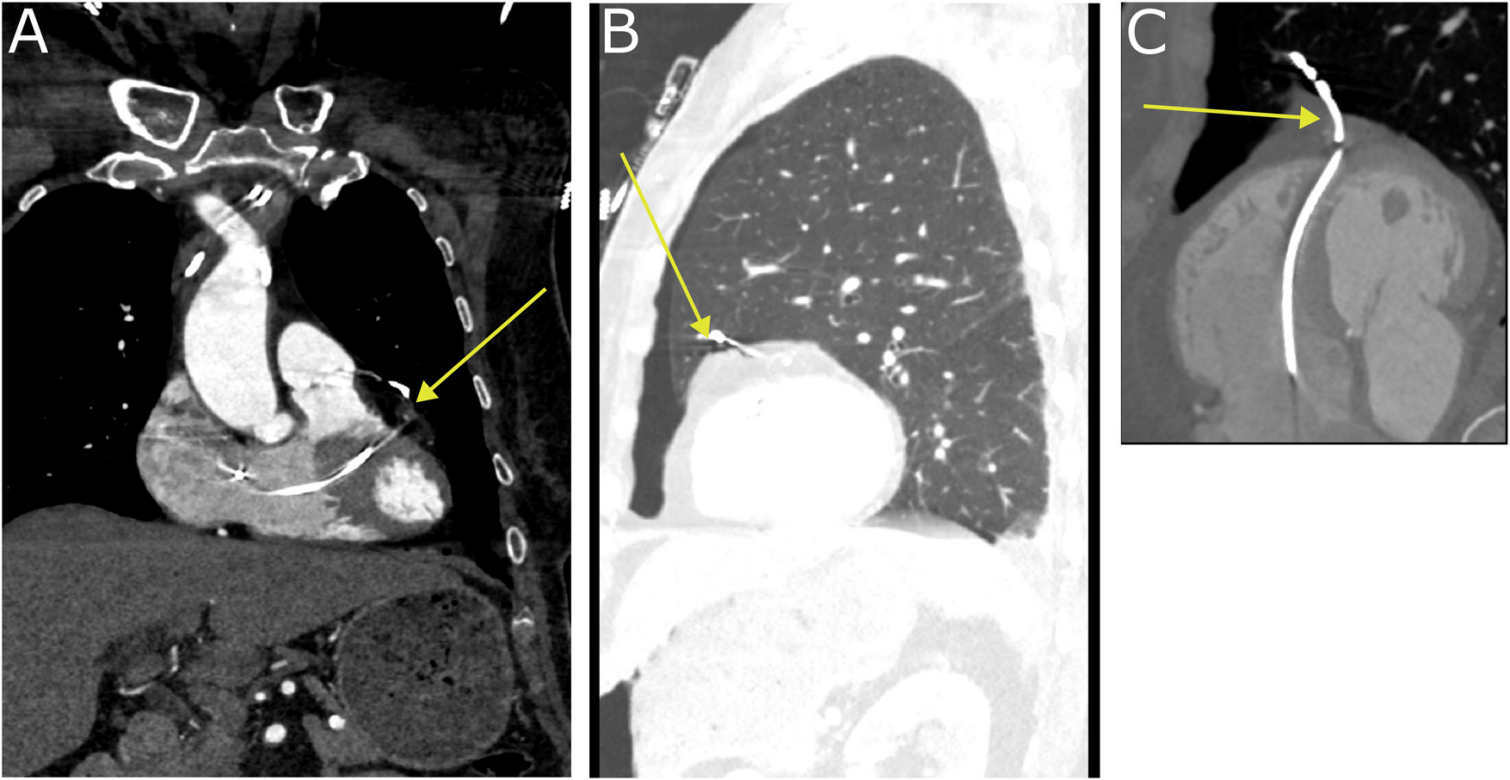
Multiplanar reconstructions from the above gated CT angiogram of the chest, showing the course of the right ventricular lead (yellow arrow) in coronal plane (A), sagittal plane (B), and along the plane of the interventricular septum (C).

She underwent a gated CT angiogram of the chest for further evaluation, which revealed that the right ventricular pacemaker lead had perforated through the interventricular septum and out through the apex of the right ventricle, passing through the myocardium, pericardium, and epicardial fat to continue through the pleural space and into the parenchyma of the left upper lobe with resulting left sided pneumothorax (Figs. 2, 3, and 4). In addition to the Cardiology service, Cardiothoracic Surgery was also consulted for removal of the perforating right ventricular lead. She was treated with supplemental oxygen through a nonrebreather mask to promote reabsorption of the pneumothorax, which spontaneously resolved 1 day later, and she remained hemodynamically stable throughout the admission.

3 days after admission, the right ventricular lead was successfully extracted transvenously in the catheter lab by the Cardiology service, with the Cardiothoracic Surgery team on standby in case of complications. The atrial lead was left in place to continue atrial pacing. The patient was closely monitored overnight in the Cardiac Care Unit. Her postoperative course was unremarkable, and she was ultimately discharged 5 days after admission.

## Discussion

We represent a rare case of subacute right ventricular perforation with extracardiac extension of lead perforation through the epicardial fat, pleura and into lung parenchyma as a complication of pacemaker lead placement in an elderly female patient.

Cardiac perforation and pneumothorax are both rare but potentially life-threatening complications of pacemaker placement, with the incidence of cardiac perforation reported as 0.4% [1] and the incidence of pneumothorax as a complication of pacemaker placement to be 1.3% [2]. Cases of atrial perforation are more common than ventricular perforation due to the thinner muscular walls of the atria as compared to the ventricles [15]. Cases of right ventricular perforation occur must commonly at the apex, which is thinner and thus more easily perforated than the interventricular septum or right ventricular outflow tract [16]. This has led to suggestions that the thicker interventricular septum or right ventricular outflow tract should be used as alternate sites of lead placement in high-risk patients [14]. As with other cases of right ventricular perforation [4,9,[11], [12], [13], [14]], in this case the perforation also occurred at the apex of the right ventricle.

Women have a higher overall risk of all complications from pacemaker placement, and higher risk of pneumothorax and cardiac perforation in particular [17]. Risk factors for cardiac perforation include older age, steroid use within 1 week of implantation, use of a temporary transvenous pacemaker, BMI<20, use of helical screw ventricular leads, and longer fluoroscopy times [18]. Risk factors for pneumothorax include age>80 years, history of COPD, dual-chamber pacemaker implantation, and subclavian venous access [19]. In this case, this patient’s risk factors for cardiac perforation and pneumothorax included her age, sex, and dual-chamber pacemaker placement.

In previous cases of right ventricular perforation, 1 case of acute right ventricular perforation was associated with contralateral pneumothorax due to extracardiac pacemaker lead migration into lung parenchyma [4], and only 1 out of 9 cases of subacute to chronic ventricular perforation [[9], [10], [11], [12], [13], [14]] was associated with contralateral pneumothorax also due to extracardiac pacemaker lead migration into lung parenchyma. This case represents a second case of subacute right ventricular perforation with associated contralateral pneumothorax as a complication of pacemaker placement.

Of note, although the pneumothorax was initially identified on chest radiograph, the right ventricular perforation was not well visualized on radiography. However, CT clearly demonstrated the cardiac perforation with extracardiac extension. Prior cohort studies suggest that CT imaging has significantly higher accuracy, sensitivity, and specificity as compared to TTE or chest radiography for diagnosis of cardiac perforation, with an accuracy of 92.9% and a sensitivity of 97%-100% as compared to an accuracy of 61.1% and sensitivity 27.7% for chest radiography and an accuracy of 62.7% and sensitivity of 41.2% for transthoracic echocardiography [20,21]. Similarly to a previous case of lead migration where cardiac perforation was not evident on radiograph and only visualized on CT [10], this case demonstrates the superior sensitivity and anatomic detail of CT compared with radiography in diagnosing cardiac perforation and defining the extent of extracardiac lead migration.

## Conclusion

Cardiac perforation with extracardiac extension into lung is a rare but potentially life-threatening complications of pacemaker placement, and the risk for these complications is higher in female patients and in the elderly. Cardiac perforation may not be well visualized on chest radiograph, and CT imaging is the gold standard in diagnosis of this rare complication. Awareness of this potential complication would allow for expedited management thereby reducing morbidity and mortality.

## Ethical statement

This study did not require institutional review board approval.

## Patient consent

Written informed consent was obtained from the patient for the publication of this article and accompanying imaging.
